# A common cognitive, psychiatric, and dysmorphic phenotype in carriers of *NRXN1* deletion

**DOI:** 10.1002/mgg3.105

**Published:** 2014-08-18

**Authors:** Marina Viñas-Jornet, Susanna Esteba-Castillo, Elisabeth Gabau, Núria Ribas-Vidal, Neus Baena, Joan San, Anna Ruiz, Maria Dolors Coll, Ramon Novell, Miriam Guitart

**Affiliations:** 1 Laboratori de Genètica, UDIAT-Centre Diagnòstic, Corporació Sanitària Parc Taulí, Institut Universitari Parc Tauli-UABSabadell, Spain; 2 Unitat de Biologia Cellular, Facultat de Biociències, Universitat Autònoma de BarcelonaBellaterra, Spain; 3 Servei Especialitzat de Salut Mental i Discapacitat Intellectual, Institut Asistència Sanitària (IAS), Parc Hospitalari Martí i JuliàGirona, Spain

**Keywords:** 2p16.3 deletion, anxiety disorder, dysexecutive syndrome, intellectual disability

## Abstract

Deletions in the 2p16.3 region that includes the neurexin (*NRXN1*) gene are associated with intellectual disability and various psychiatric disorders, in particular, autism and schizophrenia. We present three unrelated patients, two adults and one child, in whom we identified an intragenic 2p16.3 deletion within the *NRXN1* gene using an oligonucleotide comparative genomic hybridization array. The three patients presented dual diagnosis that consisted of mild intellectual disability and autism and bipolar disorder. Also, they all shared a dysmorphic phenotype characterized by a long face, deep set eyes, and prominent premaxilla. Genetic analysis of family members showed two inherited deletions. A comprehensive neuropsychological examination of the 2p16.3 deletion carriers revealed the same phenotype, characterized by anxiety disorder, borderline intelligence, and dysexecutive syndrome. The cognitive pattern of dysexecutive syndrome with poor working memory and reduced attention switching, mental flexibility, and verbal fluency was the same than those of the adult probands. We suggest that in addition to intellectual disability and psychiatric disease, *NRXN1* deletion is a risk factor for a characteristic cognitive and dysmorphic profile. The new cognitive phenotype found in the 2p16.3 deletion carriers suggests that 2p16.3 deletions might have a wide variable expressivity instead of incomplete penetrance.

## Introduction

Copy number variants (CNVs) are 1 kb or larger DNA segments that are deleted or duplicated when compared to a reference genome (Redon et al. 2006). They are found in all humans as polymorphisms. CNVs are currently emerging as an important genomic cause of disease (Rujescu et al. 2009) through disruption of genes and alteration of gene dosage. As a consequence, CNVs influence gene expression, phenotypic variation, and confer risk of complex disease traits (Redon et al. 2006). Application of array comparative genomic hybridization (aCGH) facilitates genotyping of large cohorts and the determination of new genotype–phenotype correlations. This technique has become a valuable tool for the study of mental health associated with intellectual disability (ID). Indeed, whereas the karyotype only identifies abnormalities in 5% of people with ID, aCGH increases the diagnostic yield to 11–15% (Miller et al. 2010) and up to 5–10% in autistic patients (Miller et al. 2010; Nava et al. 2014).

Clinical geneticists need to discriminate pathogenic from benign CNVs. Large studies have classified CNVs as benign variant, causative variant, or risk factor based on genetic content, function of genes included in the aberration, and CNVs inheritance pattern (Miller et al. 2010; Cooper et al. 2011; Vermeesch et al. 2012). In most studies de novo rearrangements are usually considered pathogenic, whereas inherited rearrangements from an unaffected parent are considered benign. However, some CNVs predispose to a specific phenotype with incomplete penetrance and inherited abnormalities can be significant even when phenotypes are highly discrepant between family members (Baker et al. 2012).

Genes that influence synaptic activity may contribute to different psychiatric and neurodevelopmental conditions. In a population without ID, CNVs increase susceptibility to schizophrenia and bipolar disease. In these cases, an enrichment of large (>100 kb) CNVs is observed (Malhotra and Sebat 2012). Moreover, CNVs are strongly correlated with the etiology of dual diagnosis such as ID and psychiatric disorders and/or challenging behavior. Although the prevalence of psychiatric disorders in adults with ID is higher (Deb et al. 2001; Cooper et al. 2007; Siegel and Smith 2010), the epidemiology of psychiatric disorders in the context of ID remains poorly understood because of the difficulties associated with diagnosis.

The neurexines (*NRXN*) are a group of highly polymorphic cell surface receptors that influence synaptic activity and contribute to ID and psychiatric disorders. *NRXN1* is a large gene with two independent promoters that lead the generation of longer *NRXN1-α* and shorter *NRXN1-β* extracellular variants and multiple alternative splice sites resulting in more than 3000 isoforms (Runkel et al. 2013). Different *NRXN1* transcripts interact with extracellular binding partners such as neuroligins (*NLGN*), leucine-rich repeat transmembrane neuronal protein 2, cerebellin, and dystroglycan (Missler et al. 2012). *NRXN1* binds to *NLGN* to form a calcium-dependent neurexin/neuroligin complex in the synapses of the central nervous system. This complex is crucial for an efficient neurotransmission and is involved in the formation of synaptic contacts (Reissner et al. 2008). The *NRXN1* gene is located in 2p16.3 and contains 24 exons that span 1.1 Mb with very large introns. *NRXN1* is one of the largest known human genes and it can be affected by gene disruption including missense changes, translocations, whole gene deletion, and intragenic copy number alterations with relative frequency (Ching et al. 2010). There is evidence that CNVs involving *NRXN1* are associated with cognitive ability, language development disorders, autism, and several psychiatric disorders, in particular, schizophrenia (0.47% in affected cases in contrast to 0.15% in the control population) (Bucan et al. 2009; Gratacos et al. 2009; Guilmatre et al. 2009; Need et al. 2009; Rujescu et al. 2009; Ching et al. 2010; Ikeda et al. 2010; Magri et al. 2010; Dabell et al. 2013). Results of a mouse model study suggest that deletion of the *Nrxn1* gene in mice leads to anxiety, aggression, alterations in social behavior, locomotor activity, and normal home cage behavior (Grayton et al. 2013). Most described cases have a de novo *NRXN1* deletion, but some have been inherited from an unaffected parent suggesting an incomplete penetrance.

A large series of patients with exonic deletions in 2p16.3 region and *NRXN1* mutations have been reported recently (Bena et al. 2013). The authors described developmental, neuropsychiatric, and cognitive phenotypes associated with *NRXN1* haploinsufficiency, but they did not include neuropsychological and psychiatric profiles for *NRXN1* deletion carriers. In this article, we describe three unrelated patients with a 2p16.3 deletion disrupting the *NRXN1* gene that present common dysmorphic, cognitive, psychiatric, and behavioral features. Moreover, family members who carry the deletion have been neuropsychological assessed.

## Methods

Patients were seen at the Service for Mental Health and Intellectual Disability at the Parc Hospitalari Martí i Julià (Girona, Catalunya, Spain) and referred to the Clinical Genetics Department at the Corporació Sanitària Parc Taulí (Sabadell, Catalunya, Spain) for clinical assessment. Informed consent was obtained from the patients and/or their carers.

### Clinical data

Registered variables were clinical characteristics and personal and family history. Different tests based on the intelligence level of the participants were used to evaluate psychiatric, cognitive, and behavioral disorders (see Table1).

**Table 1:** Summary of different tests used to evaluate psychiatric, cognitive, and behavioral disorders according to the level of intelligence and the age

	Cases 1 and 2: adults patients	Case 3: child	Family members
Psychopathological evaluation	PASS-ADD		TCI-R
	Compulsive behavior checklist		PAI 4
	Y-BOCS		
Cognitive evaluation	K-BIT	WISC-IV	K-BIT
	FCRO	Bayley II	FCRO
	Color Trail Test 1 and 2	Reynell	Color Trails Test 1 and 2
	PIEN-ID		WMS-III
	BRIEF		BNT
	ADOS		Semantic verbal fluency
	Tower of London		FAS
			Stroop
			BADS
			RAVLT
			Tower of London
Behavioral evaluation	ABC scale		
	ABS-RC:2		

PASS-ADD, Psychiatric Assessment for Adults with Developmental Disabilities; Y-BOCS, Yale-Brown Obsessive–Compulsive Scale; TCI-R, Temperament and Character Inventory–Revised; PAI, Personality Assessment Inventory; K-BIT, Kaufman Brief Intelligence Test; FCRO, Rey–Osterriech Complex Figure; PIEN-ID, Neuropsychological Integrated Program for people with Intellectual Disabilities; BRIEF, Behavioral Rating Inventory of Executive Function; ADOS, Autism Diagnostic Observation Schedule; WISC-IV, Wechsler Intelligence Scale for Children; WMS-III, Digits; BNT, Boston Naming Test; FAS, verbal fluency; BADS, Behavioral Assessment of Dysexecutive Syndrome; RAVLT, Rey Auditive Verbal Learning Test; ABC Scale, Aberrant Behavior Checklist; ABS-RC, Adaptive Behavior Scale – Residential and Community – Second edition.

The psychiatric diagnostic was classified according to ICD-10-ID (International Classification of Diseases – Intellectual Disabilities) and DC-LD (Diagnostic Criteria – Learning Disabilities) for the adult probands with ID and according to DSM-V for family members.

### Genetic analyses

DNA from probands and their family members was extracted from peripheral blood lymphocytes using the Gentra Puregene DNA reagent (Qiagen, Valencia, CA). Karyotype (800-G bands) and aCGH using the Agilent platform 400K (Agilent Technologies, Santa Clara, CA) were performed in all patient samples. Microarray processing was carried out according to manufacturer's specifications. For analyses of microarray data, Agilent Workbench 5.0 and Cytogenomics software (Agilent Technologies) and Nexus 6.1 (BioDiscovery, Hawthorne, CA) were used. Common CNVs (prevalence >1% in the general population) were excluded and validation of rare CNVs was performed by customized MLPA, designing specific probes according to protocols and guidelines from MRC-Holland (Amsterdam, the Netherlands) and the ProSeek web server created by Pantano et al. (2008). Once validated, family samples were also analyzed and CNVs were finally classified in three categories: pathogenic variants, benign variants, and variants of uncertain clinical significance (VOUS) (Miller et al. 2010; Vermeesch et al. 2012).

All genomic coordinates are in agreement with the UCSC Genome Browser for March 2006 (UCSC hg18, NCBI build 36).

## Results

### Molecular results

**Figure 1:**
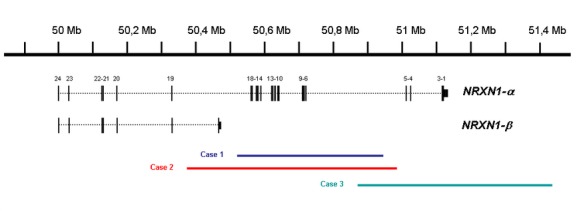
Neurexin deletion identified in case 1 (blue bar), case 2 (red bar), and case 3 (green bar). At the top of the figure is an ideogram of chromosome band 2p16.3 with genomic coordinates corresponding to the hg18 build of the human genome. Two major NRXN1 isoforms are shown in black with vertical bars representing the localization of exonic regions. 2p16.3 deletion in cases 1 and 3 affects *α* isoform while 2p16.3 deletion in case 2 affect both *α* and *β* isoforms.

The three cases presented a partial deletion in the 2p16.3 region affecting the *NRXN1* gene. Exons were removed from 5 to 18 in cases 1 and 2 and from 1 to 5 in case 3 (Fig.1). In all cases, other rare CNVs were found (Table2). The deletion was maternally inherited in cases 2 and 3. Also, two aunts of case 3 were carriers of the deletion.

**Table 2:** Summary of genotyping results

	CNVs	BP (hg18)	Length (kb)	Genes	Inheritance
Case 1	Deletion 2p16.3^1^	50,514,386–50,932,097	417	*NRXN1*	De novo
	Deletion 2q21.3	135,549,169–135,581,884	327	*RAB3GAP1*	Maternal
	Duplication 5q12.1	59,751,669–59,807,274	56	*PDE4D*	Paternal
Case 2	Deletion 2p16.3^1^	50,364,106–50,990,775	627	*NRXN1*	Maternal
	Deletion 15q22.3	63,116,991–63,149,937	33	*OSTβ RASL12*	Maternal
Case 3	Deletion 2p16.3^1^	50,894,085–51,410,728	516	*NRXN1*	Maternal
	Deletion 6q22.31	121,325,237–121,479,133	154	c6orf170	NA
	Duplication 8q13.2	70,441,952–70,587,123	145	*SUF1*	NA
	Duplication 10q25.3	117,713,715–118,044,856	331	*GFRA1*	NA

BP, break points; NA, not analyzed.

1 Common 2p16.3 deletion in the three cases.

### Clinical results

#### Case 1

**Figure 2:**
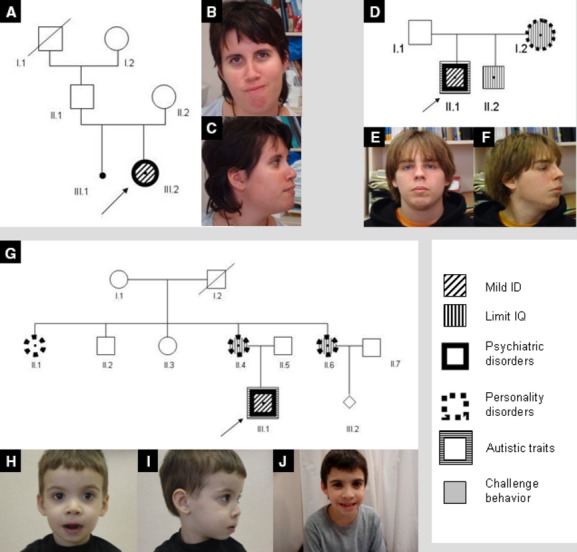
Pedigrees and photographs. (A) Case 1 pedigree; (B, C) case 1 at the age of 21; (D) case 2 pedigree; (E, F) case 2 at the age of 20; (G) case 3 pedigree; (H, I) case 3 at the age of three; (J) case 3 at the age of 11. Arrows indicate proband.

The proband (Fig.2A, III.2) was a 21-year-old female, the only child of nonconsanguineous parents. Pregnancy was uneventful and she was born full-term with a weight of 3640 g and length of 52 cm. No congenital abnormalities or feeding difficulties were observed and she started to walk at 14 months of age. The patient had febrile seizures until she was 2 years old and childhood absence epilepsy from 5 until 7 years of age. She presented difficulties in language acquisition and at the age of 12 she went to a special education school. She had a normal growth and puberty and age at menarche was 14 years. During the clinical assessment the patient presented a weight and height in the 97th centiles, an occipitofrontal circumference (OFC) of 56.5 cm (+1 SD) and good general health. Facial dysmorphism included long face, deep set eyes, hypotelorism, low set ears, prominent premaxilla, a high, narrow palate, and tooth malposition (Fig.2B and C, Table3). She also had dorsal kyphosis and long hands with slender, flexible fingers.

**Table 3:** Summary of proband's clinical data

	Case 1	Case 2	Case 3
Intellectual disability	Mild	Mild	Mild
Psychiatric disorder	Bipolar disorder	Non specified psychotic disorder	−1
Challenge behavior	+	+	+
Autistic traits	−	+	+
Dysmorphic features			
Weight	97th centiles	25th centiles	3rd centiles
Height	>97th centiles	3–10th centiles	3rd centiles
OFC	+1 SD	−1.5 SD	3rd centiles
Long face	+	+	+
Deep set eyes	+	+	+
Hypotelorism	+	+	−
Low set ears	+	+	−
Prominent premaxilla	+	+	+
High palate	+	+	−
Narrow palate	+	−	−
Tooth malposition	+	−	−
Dorsal kyphosis	+	+	−
Long hands	+	+	−
Long philtrum	−	−	+
Family history			
Family history of borderline IQ (dysexecutive pattern)	−	Mother	Mother and two aunts
Family history of psychiatric disorder (anxiety)	−	Mother	Mother and two aunts

SD, standard deviation; OFC, occipitofrontal circumference; IQ, intelligence quotient.

1 Still young to develop mental illness.

##### Psychiatric, cognitive, and behavioral profile

**Figure 3:**
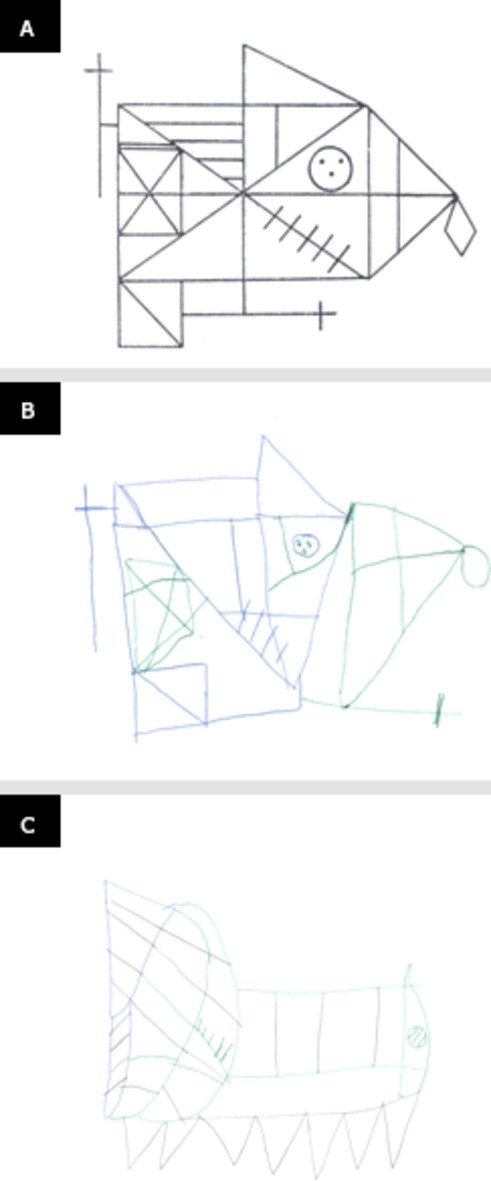
Case 1. (A) Pattern; (B) Rey–Osterriech complex figure copy; (C) Rey–Osterriech complex figure memory.

The patient had a diagnosis of bipolar disorder with a behavioral profile characterized by inappropriate reactions to frustration, which translated in refusal to follow directions, requests, or orders. Her performance on the autism diagnostic observation schedule (ADOS) was not indicative of autism spectrum disorder (ASD) or autism. Neuropsychological tests showed a mild ID with an intelligence quotient (IQ) of 65. The cognitive profile showed a dysexecutive syndrome with particularly poor behavioral control, low tolerance to frustration, and difficulty in acquiring new information, both verbal and visual (Fig.3). Language scores were within average.

The parents did not have the 2p16.3 deletion and their psychiatric and cognitive results were within normal range.

#### Case 2

The patient (Fig.2D, II.1) was a 20-year-old male with a history of ID. His mother and brother had a history of behavioral disorders. Pregnancy was uneventful; delivery was full-term and dystocic. The patient had a birthweight of 2790 g and OFC of 33.5 cm. No congenital abnormalities or feeding difficulties were observed at birth. He presented a normal growth pattern throughout childhood and puberty. At the time of assessment he had a weight of 65.5 kg (25th centile), height of 167 cm (3–10th centile), and OFC of 54 cm (−1.5 SD). Facial dysmorphism included long face, deep set eyes, hypotelorism, low set ears, prominent premaxilla, and high palate (Fig.2E and F, Table3). He presented dorsal kyphosis and finger rigidity.

##### Psychiatric, cognitive, and behavioral profile

**Figure 4:**
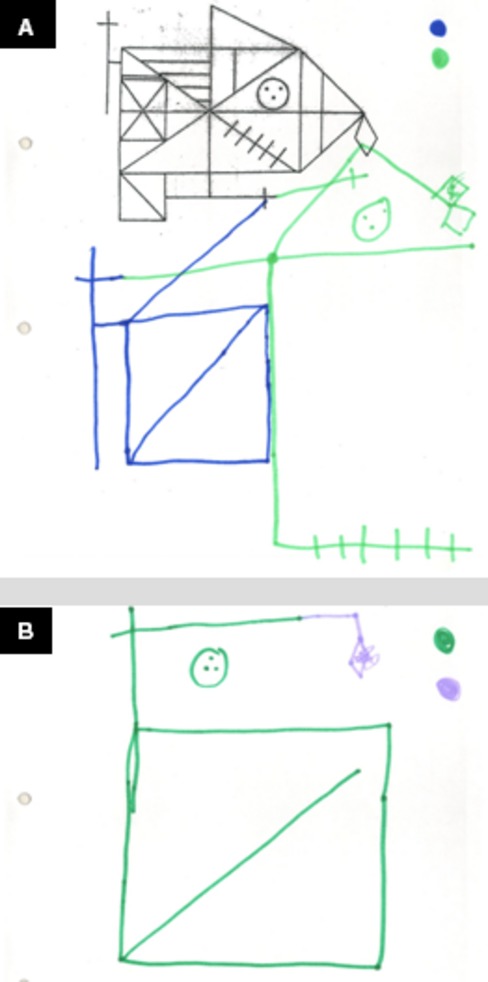
Case 2. (A) Pattern and Rey–Osterriech complex figure copy; (B) Rey–Osterriech complex figure memory.

The proband presented a nonspecified psychotic disorder with hypochondriac delusions. His behavior included explosive temper tantrums, violence, and property destruction with a diagnosis of verbal and physically aggressive destructive behavior. Within his diagnosis of atypical autism, the patient also presented obsessive–compulsive behavior. His performance on the ADOS placed him within the ASD range. Neuropsychological tests showed a mild ID with an IQ of 62 and a complex cognitive profile, which included concretism and severe impairment of executive functioning, mainly in relation to working memory, difficulty in classifying information correctly, and deficit of abstract reasoning (Fig.4).

The proband's mother, a 53-year-old housewife, was a carrier of the 2p16.3 deletion. She was schooled just for 3 years and presented a cognitive function and performance which correlated with an IQ of 69 (borderline). Her behavior was characterized by jealousy, poor self-control, suspicion, mood fluctuations, and emotional instability. She presented impulsive and anxiety traits. The neuropsychological tests revealed poor working memory and a dysexecutive pattern.

The proband's father was 51 years old. He presented a cognitive function and performance which correlated with an IQ of 97 (normal intelligence). No personality or psychiatric disorders were diagnosed.

The proband's brother was not a carrier of the 2p16.3 deletion. He was a 21-year-old man with an IQ of 76 (borderline) that performed poorly at school. He had never received psychiatric or psychological treatment.

#### Case 3

The proband (Fig.2G, III.1) was a 11-year-old boy, the only child of nonconsanguineous parents. During the pregnancy the mother suffered hyperemesis gravidarum, a surgical intervention for a sacral cyst and retroplacental hematoma. The proband was born full-term with a birthweight of 2800 g and length of 50 cm. No congenital abnormalities were observed on examination at birth, but he had breastfeeding and artificial feeding difficulties and frequent vomiting. At the age of 3, he was not able to chew, had not acquired any language, and presented severe sleep disturbances, together with maladaptive and self-harming behavior. At the age of 9, he was diagnosed of attention deficit hyperactivity disorder and cognitive delay. The patient was in a mainstream school where he followed a special program. He still presented many fears, sleep disturbances, and looked very anxious. His height, weight, and OFC had consistently been in the third centile. He also presented facial dysmorphism with a mildly long face, deep set eyes, prominent premaxilla, and long philtrum (Fig.2H–J, Table3).

##### Psychiatric, cognitive, and behavioral profile

The patient presented autistic traits, with hyperactivity and challenging behavior as his most salient psychopathological features. He had a mild ID with an IQ of 53 and a neuropsychological profile characterized by language impairment (both expression and comprehension), poor working memory, and attention. Visual reasoning performance was good.

The proband's mother was a 36-year-old woman, carrier of the 2p16.3 deletion, and the fourth of five children. She had a borderline intelligence (IQ = 75). Cognitive impairment had already been detected at primary school, where she performed poorly. The psychiatric evaluation found a generalized anxiety disorder with significant higher scores on anxiety scales (76) and anxiety-related disorders (71). A significant high score on the scale of lack of social support was obtained (74), reflecting a perception of little support. The Temperament and Character Inventory–Revised (TCI-R) results showed the same personality pattern. She had not received psychiatric or psychological support. The cognitive profile showed a dysexecutive syndrome characterized by difficulties in working memory, attention switching, mental flexibility, and verbal fluency.

The proband's father, a 42-year-old man, had an IQ of 100. He required treatment for obsessive–compulsive disorder. The cognitive evaluation of the proband's father showed a reduced verbal memory. Executive functions and visual abilities were not impaired.

Two maternal aunts (II.1 and II.6) had the 2p16.3 deletion (Fig.2G). The first carrier (II.1) had an average intelligence (IQ = 91). The scores on the Personality Assessment Inventory (PAI) were within normal range and did not highlight any clinical scale or subscale. A tendency to respond aggressively and angrily to small provocations was observed by a slight elevation in aggression subscales. The TCI-R showed a histrionic personality disorder. Her cognitive profile presented poor mental flexibility and inhibition as well as cognitive blockages. The second carrier (II.6) was a 34-year-old pregnant woman. She had performed very poorly at school. Her IQ was 86 (borderline). She scored high in anxiety (80) and on the scale of anxiety-related disorders (76), suggesting the presence of significant high levels of anxiety and stress most of the time, with fears and perseverative thoughts of catastrophe. A generalized anxiety disorder was diagnosed based on TCI-R. Cognitive problems such as difficulties in working memory, verbal and semantic fluency, and organization were detected. The uncle II.2 and the aunt II.3 (Fig.2G) did not present the 2p16.3 deletion and had an average intelligence.

## Discussion

The increasing use of the whole genome high-resolution aCGH technique in the evaluation of affected patients has identified new CNVs associated with ID, autism, and schizophrenia.

We have identified in three unrelated patients a 2p16.3 deletion including intron 5 of the *NRXN1* gene in the overlapping region, with a mean length of 520 kb (Fig.1). These deletions involve different molecular regions: the *α* isoform is affected in cases 1 and 3, whereas *α* and *β* isoforms are affected in case 2. Defects involving *NRXN1*-*β* appear to be rare compared to those involving only *NRXN1*-*α* (Duong et al. 2012; Dabell et al. 2013)Approximately 101 cases of CNVs in 2p16.3 including the *NRXN1* gene have been described, 95 deletions and 6 duplications, with a mean size of 388.97 kb (Friedman et al. 2006; Szatmari et al. 2007; Kirov et al. 2008; Zahir et al. 2008; Rujescu et al. 2009; Ching et al. 2010; Magri et al. 2010; Wisniowiecka-Kowalnik et al. 2010; Duong et al. 2012; Hedges et al. 2012; Schaaf et al. 2012; Bena et al. 2013). The *α* isoform is affected in all cases, whereas the *β* isoform is additionally affected in only 21 cases.

A recurrent phenotype with ID, language delay, motor developmental delay, ASD, and hypotonia has been described in patients affected by the *NRXN1* deletion (Bena et al. 2013). In addition, Schaaf et al. (2012) reported a larger head size and epilepsy associated to deletions in the *β* isoform, but in general dysmorphic descriptions are scarce and disparate. In this study, the three patients share some dysmorphic features: long face, deep set eyes, and prominent premaxilla. The two adult probands also showed hypotelorism, the most frequent feature described in patients with *NRXN1* deletion (Ching et al. 2010; Schaaf et al. 2012), low set ears, high palate, and dorsal kyphosis. All three patients presented dual diagnosis of mild ID and challenging behavior (child) or mild ID and mental health disease (adults). However, they did not share the same psychiatric diagnosis: bipolar disorder, nonspecified psychotic disorder with atypical autism and autistic traits in cases 1, 2, and 3, respectively. It should be noted that case 3 was still a child, and most psychiatric diseases are diagnosed from adolescence onwards. The psychiatric diagnosis of the probands is in agreement with previous observations that *NRXN1* haploinsufficiency confers risk of a wide spectrum of psychiatric diseases, including ASD, anxiety, depression, bipolar disorder, and attention deficit hyperactive disorder (Wisniowiecka-Kowalnik et al. 2010; Schaaf et al. 2012; Noor et al. 2014). The neuropsychological evaluation highlighted a characteristic cognitive pattern of dysexecutive syndrome shared by the two adults, which includes difficulties in working memory, attention switching, mental flexibility, and verbal fluency in the absence of aphasia, amnesia, agnosia, and apraxia. However, the main obstacle to their integration in supported employment programs was not their cognitive pattern but their challenging behavioral profile.

The prevalence of the *NRXN1* deletion is higher in patients compared to the control population (Schaaf et al. 2012), but rearrangements in the *NRXN1* gene are frequent in the control population (Redon et al. 2006). Indeed, the largest cohort reported *NRXN1* deletions inherited from a healthy parent in 40% of cases (Bena et al. 2013). The presence of deletions in control cases and the inheritance of this variant suggests the possibility of incomplete penetrance (Duong et al. 2012). However, no comprehensive psychological or psychiatric evaluation of carriers has been performed to date.

All participants with a 2p16.3 deletion in our study with the exception of the probands seemed unaffected at first. However, a comprehensive evaluation diagnosed various cognitive, psychiatric, and behavioral disorders. Although carrier members did not present any dysmorphic feature, they showed an IQ between 69 and 91 (borderline to average), as well as behavior and personality disorders, in particular, impulsivity and anxiety. The high levels of anxiety are in agreement with the high prevalence of anxiety traits found in the mouse model with a homozygous deletion of the *Nrxn1* gene (Grayton et al. 2013). All carriers presented a cognitive profile characterized by dysexecutive syndrome with symptoms similar to the adult probands, in the absence of aphasia, amnesia, agnosia, and apraxia. The *NRXN1* deletion affects cognitive traits in all carriers suggesting that these CNVs could have variable expressivity instead of incomplete penetrance. This hypothesis is in accordance with a recent study that conducted neuropsychological and psychiatric examinations. The neuropsychiatric CNVs carriers showed cognitive abilities between those of normal controls and ID patients (Stefansson et al. 2014). These data highlight the need for a neuropsychological and psychiatric evaluation of CNVs carriers in the normal IQ range before defining the pathogenicity of a genetic variant. The variable spectrum phenotype is an important consideration when providing genetic counseling.

The variable expressivity in deletion 2p16.2 could also be caused by additional genomic imbalances such as other rare CNVs found in some patients (Table2). Some genes influencing brain function were: *RABG3GAP1*, involved in the regulation of neurotransmitters and exocytosis of hormones; *PDE4D*, involved in various signal transduction pathways such as learning and memory; *GFRA1*, related to neuronal differentiation and survival; and *SUF1*, a growth factor regulator in early embryonic development. The interaction of different CNVs to modulate phenotypes supports theories such as the digenic/multifactorial model for neuropsychiatric diseases or the two-hit model, in which some aberrations occur in combination with other genomic CNVs (Girirajan et al. 2010; Liu et al. 2011). The additional genomic imbalances observed in our three patients may not be directly responsible for the dual diagnosis, but its effect in the phenotype should be considered.

In summary, we have described a common dysmorphic phenotype in three cases affected by a 2p16.3 deletion in addition to a common cognitive and psychiatric profile with different levels of severity among all carriers. The *NRXN1* gene deletion is a risk factor for dual diagnosis with a variable expressivity.
